# Minimal reporting guideline for research involving eye tracking (2023 edition)

**DOI:** 10.3758/s13428-023-02187-1

**Published:** 2023-07-28

**Authors:** Matt J. Dunn, Robert G. Alexander, Onyekachukwu M. Amiebenomo, Gemma Arblaster, Denize Atan, Jonathan T. Erichsen, Ulrich Ettinger, Mario E. Giardini, Iain D. Gilchrist, Ruth Hamilton, Roy S. Hessels, Scott Hodgins, Ignace T. C. Hooge, Brooke S. Jackson, Helena Lee, Stephen L. Macknik, Susana Martinez-Conde, Lee Mcilreavy, Lisa M. Muratori, Diederick C. Niehorster, Marcus Nyström, Jorge Otero-Millan, Michael M. Schlüssel, Jay E. Self, Tarkeshwar Singh, Nikolaos Smyrnis, Andreas Sprenger

**Affiliations:** 1 School of Optometry and Vision Sciences, Cardiff University, Cardiff, UK; 2 Departments of Ophthalmology, Neurology, and Physiology/Pharmacology, SUNY Downstate Health Sciences University, Brooklyn, NY USA; 3 Health Sciences School, University of Sheffield, Sheffield, UK; 4 Orthoptic Department, Sheffield Teaching Hospitals NHS Foundation Trust, Sheffield, UK; 5 Bristol Medical School, University of Bristol, Bristol, UK; 6 Department of Psychology, University of Bonn, Bonn, Germany; 7 Department of Biomedical Engineering, University of Strathclyde, Glasgow, UK; 8 School of Psychological Science, University of Bristol, Bristol, UK; 9 Department of Clinical Physics & Bioengineering, Royal Hospital for Children, NHS Greater Glasgow & Clyde, Glasgow, UK; 10 College of Medical, Veterinary & Life Sciences, University of Glasgow, Glasgow, UK; 11 Experimental Psychology, Helmholtz Institute, Utrecht University, Utrecht, The Netherlands; 12 Alysco Ventures LLP, Sedbergh, UK; 13 Department of Psychology, University of Georgia, Athens, GA USA; 14 Clinical and Experimental Sciences, University of Southampton, Southampton, UK; 15 Department of Physical Therapy, School of Health Professions, Stony Brook University, Stony Brook, NY USA; 16 Lund University Humanities Lab, Lund University, Lund, Sweden; 17 Department of Psychology, Lund University, Lund, Sweden; 18 Herbert Wertheim School of Optometry and Vision Science, University of California, Berkeley, CA USA; 19 Department of Neurology, Johns Hopkins University, Baltimore, MD USA; 20 UK EQUATOR Centre, Centre for Statistics in Medicine (CSM), Nuffield Department of Orthopaedics, Rheumatology and Musculoskeletal Sciences (NDORMS), University of Oxford, Oxford, UK; 21 Department of Kinesiology, Pennsylvania State University, University Park, PA USA; 22 2nd Department of Psychiatry, National and Kapodistrian University of Athens, Medical School, General University Hospital Attikon, Athens, Greece; 23 Department of Neurology and Institute of Psychology II, Center of Brain, Behavior and Metabolism (CBBM), University of Luebeck, Luebeck, Germany

**Keywords:** Eye movements, Eye tracking, Data quality, Reporting guidelines, Reporting standards, Reporting practices, Replicability, Reproducibility

## Abstract

A guideline is proposed that comprises the minimum items to be reported in research studies involving an eye tracker and human or non-human primate participant(s). This guideline was developed over a 3-year period using a consensus-based process via an open invitation to the international eye tracking community. This guideline will be reviewed at maximum intervals of 4 years.

**Supplementary Information:**

The online version contains supplementary material available at 10.3758/s13428-023-02187-1.

## Introduction

This guideline provides a checklist (Table 1) of minimum items to be reported by eye tracking research studies. Its purpose is to improve reporting standards to allow greater reproducibility of eye tracking research methods and to facilitate a more comprehensive understanding of the data and results presented. This list will be helpful to authors when preparing their publications, and to reviewers and journal editors when assessing the reporting completeness of submissions. The foundation for the present guideline was laid by Holmqvist et al. (2023), which comprehensively describes how the listed items can affect recorded eye tracking data.

**Table 1:** Checklist of information to include when reporting an eye tracking study

Category	Item no.	Checklist item	Reported on page no.
Items to be reported by all eye tracking studies	A1	Manufacturer and model	
	A2	Software and firmware versions	
	A3	Eye tracking technology	
	A4	Sampling frequency	
	A5	Head movement restrictions	
	A6	Eye(s) recorded	
	A7	Parameters recorded	
	A8	Environment lighting	
	A9	Calibration	
	A10	Measurement uncertainty	
	A11	Data processing steps	
	A12	Data loss	
Additional item to be reported by studies of eye movement dynamics	B1	Signal latencies	
Additional item to be reported by studies reporting screen-based gaze coordinates	C1	Participant to display monitor distance	

### Scope of the guideline

The guideline applies to any study which involves an eye tracker and human or non-human primate participant(s). Since the expertise of the author group lies primarily in human and non-human primate work, the guideline is aimed at research with only these species. While the guideline may prove helpful when designing research studies and preparing manuscripts involving studies of other species, the eye tracking technology involved may be considerably different, so judgement must be made on a case-by-case basis as to whether the guidance presented here is applicable in these settings.

*Eye tracking* is defined as the measurement of eye or gaze direction or the movement over time or the parameters derived from the data obtained, by an instrument referred to as an *eye tracker*. The measurement parameters are defined in detail in the checklist items.

The guideline is designed to be applicable to all currently available forms of eye tracking technology, including but not limited to camera-based eye trackers, systems relying on infrared reflectance, and electrode-based systems. The guideline will be revised and updated at regular intervals and as new technology emerges (see Guideline review and update process).

Incidental measurements that eye trackers make (such as head position, pupil size, or blinks) are outside the scope of this guideline because they are not eye tracking per se. Note that suitable guidance already exists for pupillometry (Kelbsch et al., 2019). This reporting guideline provides a minimum list of reportable items relating specifically to eye tracking. It therefore does not replace other reporting guidelines that cover broader aspects of study design (e.g., CONSORT for randomized trials, STROBE for observational studies, etc.), nor does it repeat items expected to be reported in intersecting research fields (such as stimulus size, which should be reported in vision research).

### Flexibility

Reporting guidelines set the minimum amount of information needed in a scientific article for readers to understand, critically appraise, and reproduce the methods. However, the extent to which reporting can be considered incomplete depends on the nature of any given study. We therefore advise against rigid adoption of the guideline as an inflexible barrier to publication. All items in the guideline should be *considered*, but failing to report an item should not automatically preclude publication. Authors, editors, and reviewers should consider on a case-by-case basis whether unreported items have the potential to undermine reproducibility or interpretability in the context of the particular study. For more in-depth considerations, we refer the reader to Holmqvist et al. (2023).

### Terminology

The terminology employed in this document aims to follow established ISO 99:2007 norms (International Organization for Standardization [ISO], 2007). However, note that, owing to consistent historical usage by the eye tracking community, the term ‘calibration’ is used with a looser meaning and refers to any procedure that enables an eye tracker’s output to be interpreted within its intended scope, i.e., to assess the empirical validity (Peacock & Peacock, 2010, p. 94) of the measured parameters.

## Methods

The guideline was developed over a 3-year period by researchers and clinicians representing numerous eye tracking sub-specialties. The initiative was advertised internationally via e-mail lists and clinical/research groups from December 2019 to May 2020. Group membership was open to anyone with an interest in helping to develop the guideline. The initiative was a horizontal community effort with no individual or group leading the process; all decisions were made by the majority present at each meeting. A minimum of six active participants were required for meetings to be deemed quorate. Twelve quorate meetings were held between May 2020 and February 2023, with the number of participants ranging from eight to over 23. Representatives from the EQUATOR Network attended four of the meetings to provide advice and ensure the process followed current recommendations for reporting guideline development (Moher et al., 2010). All meetings were held online.

In May 2020, a meeting was held to discuss whether an eye tracking methods reporting guideline was needed, and consider whether existing published guidance (Hessels & Hooge, 2019; Oakes, 2010) was sufficient to provide a minimum reporting guideline covering most eye tracking research applications. The group decided that a complete guideline was warranted and opted to produce a single document to cover minimum reportable items across all eye tracking fields, rather than separate guidelines for each subspecialty, since most issues affecting eye tracking data quality are common across research fields. Following the *Delphi Method* (Brown, 1968), from January to March 2022, all 47 members of the group were invited to contribute anonymously to an unstructured list of items to be considered for inclusion, resulting in 69 suggestions (see Supplementary Material for list). In September 2022, a structure was added to the guideline to enable inclusion of items specific to certain types of eye tracking studies. From September to December 2022, over three rounds, each item on the preliminary list was discussed in the context of the guideline scope and its inclusion was voted on by the group to reach the final checklist presented in Table 1 of minimum reportable items.

## Checklist items

Below, we provide the rationale and an example of ideal reporting for each item included in the checklist. For some items (A3, A7, and A12), constructed examples are given, as we were unable to find any suitable publication to cite as an example of good reporting for these items. Note that some studies might require more or fewer details when reporting their methods. The checklist and worked examples provided here have the aim of reminding authors about the minimum aspects of their research that must be described in their publications to allow critical appraisal and reproducibility.

### Items to be reported by all eye tracking studies

#### Item A1: Manufacturer and model

**Example** – “To track eye movements, participants wore a pair of SMI Eye Tracking Glasses […] (SMI GmbH, Berlin, Germany)” (Sullivan et al., 2021).

**Explanation** – Identifying both the manufacturer and model enables the reader to seek further information about the eye tracker used in the study. Supplying the city and country helps the reader to locate the manufacturer in order to replicate a study using similar hardware. If the eye tracker used in the study is self-built or not commercially available, a complete description of the eye tracker design should either be included or referred to in a readily accessible supplement.

#### Item A2: Software and firmware versions

**Example** – “This setup used the Tobii Pro Glasses 2 […] (firmware version 1.25.3-citronkola). […] This setup used the […] headset in combination with the open-source eye tracking software EyeRecToo (version 2.0 commit 51a839aa).” (Niehorster et al., 2020)

**Explanation** – Software and firmware are programs required to run most eye tracking hardware. Since they are used alongside eye tracking devices, the version number typically defines the inherent functionality and how recent or up to date the eye tracking system is. Software and firmware are used during the calibration process, to record data, present stimuli, process and analyze the data. If the recording process was controlled by bespoke software, this should be described in sufficient detail (or the code provided if possible).

#### Item A3: Eye tracking technology

**Example** – “We used the […] eye tracker, a video-based eye tracker providing gaze coordinate data”.

**Explanation** – There are many different methods for tracking eye movements or gaze direction. These methods include, but are not limited to: electrooculography, scleral coils, dual Purkinje imaging, limbus tracking, video-oculography, and retinal-image-based tracking. See Holmqvist et al. (2023) for details on these methods. The nature of the eye tracking signal should be reported, i.e., whether the system natively provides data as, e.g., an analog voltage, digital gaze coordinates, or digital gaze directions.

#### Item A4: Sampling frequency

**Example** – “Eye movements were recorded [...] at a sample rate of 250 Hz" (Jayaramachandran et al., 2014).

**Explanation** – Sampling frequency (the number of times per second the tracking data is recorded, expressed in Hertz) is an indicator of the eye tracker’s ability to represent time-dependent parameters, e.g., fixation duration, eye velocity and acceleration, and the onset and termination of events such as fixations and saccades. The relevance of sampling frequency depends on the phenomena under investigation, e.g., the evaluation of saccadic peak velocity is typically much more sensitive to sampling frequency than that of fixation duration (Mack et al., 2017). If the eye tracker samples at irregular intervals, this must be stated.

Note that this item refers to native sampling frequency. If the data are resampled to a different frequency, this should be specified as a data processing step (see Item A12).

#### Item A5: Head movement restrictions

**Example** – “The participant's head was supported on a chin-rest” (Murray et al., 2022).

**Explanation** – It should be stated whether the head movement was free (unrestrained) or whether head movement was restrained in any way. Head restraints may include but are not limited to: chin rest, forehead rest, bite bar, or a child may be seated on their parent’s knee with the head held by the parent. If the head is unrestrained, the extent to which head movements could impact data reliability should be considered and reported.

#### Item A6: Eye(s) recorded

**Example** – “Eye positions were recorded for both eyes” (Ukwade & Bedell, 1992).

**Explanation** – It should be stated whether the parameters measured were from one eye (and if so, which eye) or both eyes. Please note that this item refers to the data recorded. Whether a parameter is measured through a process that involves one or both eyes (e.g., a gaze direction measured through monocular or binocular eye tracking) is information related to the eye tracking technology, to be reported as part of Item A3.

#### Item A7: Parameters recorded

**Example** – “The eye tracker recorded horizontal and vertical gaze position in pixels on the screen, where (0,0) corresponds to the top-left of the screen.”

**Explanation** – The parameters measured by the eye tracker and recorded for subsequent interpretation should be listed. To allow an unequivocal interpretation of these parameters, the coordinate system used should also be reported, including its units (e.g., degrees, pixels, etc.), frame of reference (e.g., world-centered coordinates such as a position on a screen/plane/object, head-centered etc.), origin (zero), and (where necessary to interpret the presented data) the directions (e.g., up/down) represented by positive and negative values.

#### Item A8: Environment lighting

**Example** – The […] environment was a room with large windows facing the tracker; additional lighting came from […] fluorescent lights. Data was collected on a cloudy day” (Feit et al., 2017).

**Explanation** – Environment lighting may affect eye tracking data quality. At the least, a statement indicating whether the environment was dark, moderate, or light should be provided, referring to a reference/definition for these terms where meaningful.

#### Item A9: Calibration

**Example** – “Rather than using the built-in EyeLink 1000 calibration sequence, a custom 5-point (0°, ±10° vertical, ±10° horizontal presented for 4 s each at location) monocular calibration was performed for each eye by fixating a small white dot (0.3° diameter) presented on a black background, and validated using a four-point validation procedure (average validation error 0.8°)” (example based on Kelly et al. (2019), Niehorster et al. (2019)).

**Explanation** – In the majority of published eye tracking research, the calibration process is a key determinant of the interpretation of the parameters measured. If the eye tracker was not subject to a formal calibration process, this should be stated. If the eye tracker was calibrated, the following should be reported:

1. **Calibration method**: State whether the calibration process was provided by the eye tracker manufacturer, or if an alternative method was used – in which case, sufficient detail should be provided to replicate it.
2. **Calibration design**: Details about the design of the calibration process, including stimulus, the number of calibration targets used, their color, size, shape, duration and position or movement should be provided. A statement describing any calibration-related participant task should be made (e.g., *press a button when looking at a target*), as this may affect the significance of the calibration outcome (e.g., people with motion impairment may take longer to press a button in response to a stimulus than people with normal movement).
3. **Calibration validation**: The criteria against which the calibration was accepted as valid should be provided. If a validation procedure was performed, the validation method should be described using the same guidelines as for calibration method and design. If the quality of the calibration was monitored during the recording, with drift checks or by other means, this should be stated. If these checks may result in recalibration being performed, this should be stated, and the criteria to trigger a recalibration should be described.

#### Item A10: Measurement uncertainty

**Example** – “Precision in terms of sample-to-sample RMS distance of the gaze data, averaged across participants, was 0.30°, and in terms of mean standard deviation, 0.42°. These values did not differ between conditions.” (Niehorster et al., 2019)

**Explanation** – For any quantities reported, the estimated uncertainties should be stated. These may be reported using any measure suitable for the specific experimental context. E.g., when reporting precision of gaze position, this is typically reported as root mean square, sample-to-sample deviation (RMS-S2S), standard deviation or bivariate contour ellipse area.

#### Item A11: Data processing steps

**Example** – “Any samples reporting a gaze position ≥ 50% beyond the screen edge were discarded as artefacts. Short gaps in the data (≤ 25 ms) were interpolated with cubic splines. Any remaining data lying ≥ 10 standard deviations from the median gaze position for the entire recording were discarded as artefacts. Next, to remove remaining blink-related artefacts, all data ≤ 75 ms either side of all gaps in the data were also deleted. Position data were then filtered using a generalized Savitzky–Golay filter […] Saccades were detected using the method described by Engbert and Kliegl” (Cutsuridis et al., 2021).

**Explanation** – All data processing steps in eye tracking include manipulations to the native eye tracker signal as reported in Item A7, to produce the parameters relevant to the experimental study (e.g., to convert the native signal into a gaze signal or a velocity signal, fixation duration, saccade amplitude, blink rate, etc.). These steps may be performed by commercial or custom software. See Figure 1 of Holmqvist et al. (2023), for an outline of data processing steps involved in eye tracking.

#### Item A12: Data loss

**Example** – “18% of samples were lost, 5% of which were as a result of participant blinks. These samples appeared randomly distributed across participants, conditions, and time”.

**Explanation** – Data loss refers to the proportion of samples either reporting no tracking data, or which were discarded during analysis as not being representative of measured data. E.g., let us assume that a 250-Hz eye tracker is expected to deliver 250 gaze coordinates per second. If only 225 reliable samples are delivered, 10% data loss is observed. Data loss may result from experimental conditions, such as blinks, or from an issue relating to the eye tracker or participant setup and may be reported in different ways depending on the eye tracker and purpose(s) of the study. If the eye tracker has a fixed sampling frequency, this should be expressed as a percentage; if not, the effective frequency should be reported (see Hooge et al. (2022) for considerations regarding which measure to use).

### Additional item to be reported by studies of eye movement dynamics

The item below should be reported where relevant, especially for studies employing gaze-contingent stimulus presentation, or in which multiple temporal signals are combined across different technologies (e.g., neuroimaging studies). This item may also be important in eye tracking studies that are concerned with the latency, speed, or other dynamic property of eye movements (e.g., studies of pursuit or saccadic reaction times) where synchronization between the eye tracker and stimulus delivery system is required for accurate estimates of such parameters.

#### Item B1: Signal latencies

**Example** – “Updating the display contingent on the viewer’s gaze required 1 ms to receive a sample from the eye tracker, less than 1 ms to draw the three image textures, and up to 7 ms to refresh the screen” (Nuthmann, 2014).

**Explanation** – System latency refers to the duration of a signal traveling from the input to the output of a system. For a video-based eye tracker, this is the time taken to produce gaze coordinates from the light reaching the eye tracker camera. For a gaze-contingent display (a system in which gaze is actively used to manipulate a stimulus), the total system latency includes eye tracker latency and the latency of the stimulus presentation system. Systems with long and varying latencies may produce signals that are problematic to interpret. When an experimental setup consists of several subsystems (e.g., concurrent eye tracking, head tracking, and EEG), the different subsystem latencies may complicate synchronization. Any latency (device latencies, system latencies, etc.) should therefore be reported if likely to be relevant with reference to the experiment rationale and design.

### Additional item to be reported by studies reporting screen-based gaze coordinates

The item below should be reported in any eye tracking study in which gaze coordinates are defined relative to a display monitor.

#### Item C1: Participant to display monitor distance

**Example** – “Observers were measured at a distance as close to 0.620 m as possible and our software blanked out the screen and displayed a warning message (which suspended data acquisition) whenever the observer’s eyes were closer than 0.520 m or further than 0.720 m from the screen. The stimulus was presented on a screen in a fronto-parallel plane at a distance of 0.5 m from the participant.” (Mooney et al., 2021)

**Explanation** – When a display monitor is used as the reference frame for gaze coordinates, the distance of the eye(s) to the display monitor and its dimensions should be reported. This allows verification of the proportion of the monitor falling within the eye tracker’s trackable range and allows the reader to determine whether the recorded gaze directions contain a significant vergence component.

The plane of stimulus presentation should also be reported if there is a significant vergence component of eye movements. E.g., if the stimuli are presented on a screen lying flat on a table top, vergence eye movements will change for targets presented at different depths.

## Guideline review and update process

Timing of updates of this document will be guided by feedback and use as well as technological and other developments in the field. Review will occur at maximum intervals of 4 years.

## Conclusion

This guideline presents a recommended minimal set of items to consider reporting in eye tracking studies. The aim is to improve reproducibility of methods and comparability of results.

### Supplementary Information

Below is the link to the electronic supplementary material.

Supplementary file1 (PDF 30 KB)

## Data Availability

Not applicable.

## Code availability

The latest version of the guideline is available on GitHub at https://github.com/dcnieho/ET_reporting_guideline.
