# Author Correction: Minimal reporting guideline for research involving eye tracking (2023 edition)

**DOI:** 10.3758/s13428-024-02438-9

**Published:** 2024-04-30

**Authors:** Matt J. Dunn, Robert G. Alexander, Onyekachukwu M. Amiebenomo, Gemma Arblaster, Denize Atan, Jonathan T. Erichsen, Ulrich Ettinger, Mario E. Giardini, Iain D. Gilchrist, Ruth Hamilton, Roy S. Hessels, Scott Hodgins, Ignace T. C. Hooge, Brooke S. Jackson, Helena Lee, Stephen L. Macknik, Susana Martinez-Conde, Lee Mcilreavy, Lisa M. Muratori, Diederick C. Niehorster, Marcus Nyström, Jorge Otero-Millan, Michael M. Schlüssel, Jay E. Self, Tarkeshwar Singh, Nikolaos Smyrnis, Andreas Sprenger

**Affiliations:** 1 School of Optometry and Vision Sciences, Cardiff University, Cardiff, UK; 2 Departments of Ophthalmology, Neurology, and Physiology/Pharmacology, SUNY Downstate Health Sciences University, Brooklyn, NY USA; 3 Health Sciences School, University of Sheffield, Sheffield, UK; 4 Orthoptic Department, Sheffield Teaching Hospitals NHS Foundation Trust, Sheffield, UK; 5 Bristol Medical School, University of Bristol, Bristol, UK; 6 Department of Psychology, University of Bonn, Bonn, Germany; 7 Department of Biomedical Engineering, University of Strathclyde, Glasgow, UK; 8 School of Psychological Science, University of Bristol, Bristol, UK; 9 Department of Clinical Physics & Bioengineering, Royal Hospital for Children, NHS Greater Glasgow & Clyde, Glasgow, UK; 10 College of Medical, Veterinary & Life Sciences, University of Glasgow, Glasgow, UK; 11 Experimental Psychology, Helmholtz Institute, Utrecht University, Utrecht, The Netherlands; 12 Alysco Ventures LLP, Sedbergh, UK; 13 Department of Psychology, University of Georgia, Athens, GA USA; 14 Clinical and Experimental Sciences, University of Southampton, Southampton, UK; 15 Department of Physical Therapy, School of Health Professions, Stony Brook University, Stony Brook, NY USA; 16 Lund University Humanities Lab, Lund University, Lund, Sweden; 17 Department of Psychology, Lund University, Lund, Sweden; 18 Herbert Wertheim School of Optometry and Vision Science, University of California, Berkeley, CA USA; 19 Department of Neurology, Johns Hopkins University, Baltimore, MD USA; 20 UK EQUATOR Centre, Centre for Statistics in Medicine (CSM), Nuffield Department of Orthopaedics, Rheumatology and Musculoskeletal Sciences (NDORMS), University of Oxford, Oxford, UK; 21 Department of Kinesiology, Pennsylvania State University, University Park, PA USA; 22 2nd Department of Psychiatry, National and Kapodistrian University of Athens, Medical School, General University Hospital Attikon, Athens, Greece; 23 Department of Neurology and Institute of Psychology II, Center of Brain, Behavior and Metabolism (CBBM), University of Luebeck, Luebeck, Germany

**Author Correction: Behavior Research Methods**

10.3758/s13428-023-02187-1

The original online version of this article was revised. The following funding note was added:

This work was supported by the New York State Empire Innovator Program, by the National Science Foundation (Award 1734887 to SM-C and SLM; Award 1523614 to SLM), and by the National Institute of Health (Award R01EY031971 to SM-C and SLM; Award R01CA258021 to SM-C and SLM). The content is solely the responsibility of the authors and does not necessarily represent the official views of the National Science Foundation nor the National Institutes of Health.

