# Supplementary material for: Minimal reporting guideline for research involving eye tracking (2023 edition)
Source: Behav Res Methods. 2023 Jul 28;56(5):4351–7. doi: 10.3758/s13428-023-02187-1 (PMC11225961; doi:10.3758/s13428-023-02187-1)
Supplement: Supplementary file 1 — Supplementary file1 (PDF 30 KB) [file 13428_2023_2187_MOESM1_ESM.pdf]

Supplementary material: initial item list

The list below contains all 69 anonymous suggestions originally proposed by the group for inclusion in the guideline. Please note that **this is not the final checklist**; it is presented solely to illustrate the guideline development process. The final checklist can be found in Table 1 of the manuscript.

Group A (Environment)

- Illuminance
- Vibrations
- Infrared light sources
- Ambient noise
- Presence of others

Group B (Eye tracker)

- Manufacturer (including city and country)
- Model name
- Technique
- Filters (built-in)
- Tracking volume
- Soft- and firmware versions

Group C (Visual stimulus)

- Dimensions
- Luminance (max, min, Gamma corrected)
- Chromatic properties
- Uniformity (of luminance / chromatic pr.s)
- Frame Rate

- Contrast
- Other properties
- Source
- Participant to eye tracker distance
- Participant to display monitor distance
- Head movement restrictions

Group D (Participant characteristics)

- Age
- Gender/Sex
- Visual acuity
- Visual aids / corrections
- Makeup
- Eye colour and specific iris features
- Baseline pupil size
- Interocular distance
- Palpebral fissure (eye cleft)
- Eye-lash direction
- Eye dominance
- Eye recorded
- Facial movements; due to speech etc.
- Sleep deprivation
- Expertise
- Oculomotor atypicalities
- Clinical characteristics
- Consent
- Substance use (state effects)

- Knowledge of the hypothesis

Group E (Calibration)

- Target
- Number/position of calibration points
- Number/position of validation points
- Binocular or monocular
- Control
- Operator expertise
- Drift compensation and recalibrations

Group F (Experiment)

- Trials
- Practice trials
- Instruction
- Recording
- Breaks
- Exclusion criteria
- Frequency of validation

Group G (Signal)

- Sampling frequency
- Latency
- Data loss
- Attrition rate
- Spatial accuracy
- Spatial precision

Group H (Events and AOIs)

- Name of event detection algorithm
- Algorithm thresholds
- Area of interest size
- Area of interest margin
- Distances between AOIs
- Area of interest overlap
- Sample or event-based area of interest metrics
